# Clinical and Radiographic Success of Pulpotomy with MTA in Primary Molars: 30 Months Follow up

**Published:** 2010-11-15

**Authors:** Roza Haghgoo, Farid Abbasi

**Affiliations:** 1. Department of Pedodontics, Dental School, Shahed University of Medical Sciences, Tehran, Iran.; 2. Department of Oral Medicine, Dental School, Shahed University of Medical Sciences, Tehran, Iran.

**Keywords:** Radiographic Image, Pulpotomy, ProRoot MTA, Primary Tooth, Root MTA

## Abstract

**INTRODUCTION:**

Pulpotomy of carious primary teeth with an exposed pulp is a common treatment option. Pulpotomy has been conducted with various medicaments over the years. The aim of this study was to evaluate clinical and radiographic success of primary vital pulpotomy with ProRoot and Root MTA.

**MATERIALS AND METHODS:**

In this randomized clinical trial, children aged between 3-7 years who met the inclusion criteria were enrolled. A total of 70 teeth were deemed suitable under the inclusion criteria and teeth were randomly divided into the 2 groups; ProRoot and Root MTA. Pulpotomy was performed and immediately followed by coronal amalgam restoration. The clinical and radiographic follow ups were conducted 6, 12, 18, 30 months post-operatively. The data were analyzed using Exact Fisher test.

**RESULTS:**

At the final follow up, 28 teeth in ProRoot MTA and 26 teeth in Root MTA were evaluated. In the Root MTA group, 1 tooth had exfoliated and one had an abscess and furcal radiolucency radiographically. In ProRoot MTA group, external resorption was observed in 1 tooth. Statistical analysis did not show significant difference in success rate between 2 groups after 30 months.

**CONCLUSION:**

The success rates of Root and ProRoot MTA are similar, indicating that pulpotomy can be carried out successfully in both primary molars.

## INTRODUCTION

The objective of pulpotomy treatment of a primary molar is to preserve the tooth until natural exfoliation and eruption of permanent successor occurs. Pulpotomy treatment consists of coronal pulp removal, placement of medicament and the final permanent restoration [1][2]. Several agents have been used in pulpotomy, including formecresol and mineral trioxide aggregate (MTA) [3][4][5][6][7]. MTA was introduced by Torabinejad and is composed of tricalcium silicate, tricalcium aluminate and tricalcium oxide and silicate oxide. Hydration of MTA produces a colloidal gel that sets gradually as the pH reaches 12 [1][2]. MTA has low cytotoxicity, good biocompatibility [3] and is also antibacterial to a degree. Furthermore, it can preserve tissue integrity more than calcium hydroxide [8][9][10] and can induce hard tissue formation [8][11][12][13]. These characteristics make MTA a suitable material for pulpotomy [8][9][10][11][12][13][14][15]. Success rates of pulpotomy with MTA have been evaluated in several studies. Some studies suggest that there is no significant difference between success rate of MTA and formecresol, though others have caused controversy by suggesting either higher success rates for MTA or formecresol [16][17][18][19]. Root MTA has been introduced (Tabriz, Iran) as an alternative to ProRoot MTA and showed similar characteristics to ProRoot MTA in histological and in vitro studies. These studies revealed that the cytotoxicity of Root MTA on L929 is less than ProRoot MTA [20] and that there are no significant difference between Root MTA, ProRoot MTA and Portland cement when evaluating tissue inflammatory response, fibrous capsule and bone formation [21].Results of a further clinical study revealed that the difference in success rate of pulpotomy with Root MTA, ProRoot MTA after 12 months is not statistically significant [22].

The aim of this study was to compare the long term clinical and radiographic success rate of pulpotomy with Root MTA and ProRoot MTA.

## MATERIALS AND METHODS

This randomized clinical trial was performed on children who were referred to pediatric department of Shahed Dental School for dental treatment. Inclusion criteria for subjects were 1) age range of 3-7 years; 2) primary molar in need of pulpotomy; 3) healthy systemic status with no contraindication for pulpotomy; 4) carious primary molar teeth that required pulpotomy. Written informed consent was obtained from their parents. The clinical criteria for tooth selection were 1) no clinical symptoms such as spontaneous pain during the night; 2) no tenderness to pressure/percussion; 3) no mobility; 4) absence of associated swelling or sinus tract; 5) absence of vital carious pulp exposure on examination; and 6) presence of restorable crowns. Radiographic criteria included the absence of: 1) internal and external resorption; 2) furcal radiolucency; and 3) root canal calcifications. Seventy teeth, in accordance with similar studies [6][15][22], that met the inclusion criteria were randomly allocated in either Root MTA (Tabriz, Iran) or ProRoot MTA (DENTSPLY Tulsa), (ProRoot group code 0, Root MTA group code 1). Randomized allocation was conducted by dental assistant who was blinded to the study design. The primary tooth pulpotomies were performed by the same pedodontist throughout the study. After anesthesia, caries was removed and coronal access was made with a #245 bur (Dentsply Maillefer, Tulsa, OK, USA) and high speed hand piece. Coronal pulp was removed with a spoon excavator; subsequently, the pulp chamber was irrigated with saline. Hemorrhage was controlled by placing a cotton pellet moistened in saline with slight pressure. Once hemostasis was achieved in both groups the material were mixed according to instructions and placed within the pulp chamber. All teeth were restored with amalgam and examined after 6, 12, 18 and 30 months clinically and radiographically by a blind dentist. Clinical and radiographic success criteria were as follows: 1) the absence of pain, mobility, swelling, and sinus tract; and 2) radiographic absence of internal and external resorption and furcal radiolucency [23][24][25]. The presence of even single clinical and/or radiographic failure rendered the treatment as a failure. Data were analyzed using Fisher Exact test.

## RESULTS

At the 6 month post-operative evaluation, 34 teeth in Root MTA group and 33 teeth in ProRoot MTA group were examined. No failure was observed in the 6-month samples. In the 12 months follow up, 31 teeth in ProRoot and 31 teeth in Root MTA group were evaluated. In Root MTA group, all the cases showed successful results. In ProRoot MTA group, two failures were observed including an abscess and furcal radiolucency. Fisher Exact test revealed no statistically difference in success rate between ProRoot and Root MTA. The 18-month follow up was performed on 30 cases in ProRoot MTA and 31 cases in Root MTA group which were all successful. During the 30 months post operative evaluation, 28 teeth in ProRoot MTA and 26 teeth in Root MTA groups were available for assessment. In the Root MTA group, one tooth exfoliated and one demonstrated abscess and furcal radiolucency (as well as canal calcification which impeded pulpectomy treatment) at the 30 month follow up evaluation (Table 1, Table 2). In ProRoot MTA external resorption was seen in 1 tooth; the difference between two groups was not statistically significant.

**Table 1:** Radiographic signs 30 months after pulpotomy with Root MTA and ProRoot MTA

**Materials**	**N**	**IR^a^**	**ERb**	**FRc**
**Root MTA**	26	0	0	1(3.8%)
**ProRoot MTA**	28	0	1(3.5%)	0

^a^ IR: Internal resorption

^b^ ER: External resorption

^c^ FR: Furcal radiolucency

**Table 2:** Clinical signs 30 months after pulpotomy with Root MTA and ProRoot

**Clinical signs**	**Pain**	**Swelling**	**Mobility**	**Sinus tract**	**Tenderness to percussion**	**Abscess**
**Root MTA[26]**	0	0	0	0	0	1(3.8%)
**ProRoot MTA[28]**	0	0	0	0	0	0

## DISCUSSION

Pulpotomy is a common treatment modality in primary teeth with carious exposed pulp. This procedure preserves the tooth and the arch space for permanent teeth; thereby avoiding future problems [26][27]. Formecresol is the common medicament in pulpotomy; it has some distinct disadvantages such as cytotoxicity and potential of mutagenicity and carcinogenicity. [28][29].MTA is biocompatible and antibacterial material with little cytotoxicity. Moreover, it induces cell proliferation and regeneration [3][4][5][8][9], and has been proposed as a suitable medicament for pulpotomy. Root MTA has been produced in Iran and has the same favorable characteristics of ProRoot MTA [20][21][22]. In this study, we evaluated the long-term success rate of pulpotomy of primary molars with Root MTA and ProRoot MTA. Results of this study showed high radiographic (Root MTA=96.16% and ProRoot MTA=100%) and clinical (Root MTA=96.16% ProRoot MTA=96.43%) success. The difference between two materials was not statistically significant.

Ramezankhani et al. and Sadre Lahigani's et al. demonstrated that the inflammation and biocompatibility of ProRoot MTA and Root MTA was not different, possibly due to their similar characteristics and composition [21][30].

A previous study reported 100% clinical and radiographic success rates for pulpotomy with Root MTA and 96.78% success rates for pulpotomy with ProRoot MTA after 1 year, concurring with our study [22]; however, our study consisted of longer follow up period.

Only one tooth demonstrated periapical infection and root canal calcification impeding further treatment in the present study. This may only pose a problem to treatment outcome if a significant percentage of pulpotomized primary teeth demonstrated root calcifications and associated abscesses following treatment.

In this study, clinical and radiographic success rate of ProRoot and Root MTA was favorable after 30months.

## CONCLUSION

ProRoot MTA and Root MTA can be considered suitable materials for pulpotomy of primary molars. Further studies should address and compare the histological success rates of ProRoot and Root MTA.
